# Construction and validation of a model for creating simulated scenarios based on Jeffries Theory

**DOI:** 10.1590/0034-7167-2024-0222

**Published:** 2025-08-08

**Authors:** Jéssica de Oliveira Veloso Vilarinho, Marcia Bucco, Ana Elizabeth Lopes de Carvalho, Nilton Orlando da Silva, Luciana Puchalski Kalinke, Jorge Vinícius Cestari Felix

**Affiliations:** I Universidade Federal do Paraná. Curitiba, Paraná, Brazil

**Keywords:** Psychometrics, Simulation Training, Validation Study, Nursing, Health Education., Psicometría, Entrenamiento Simulado, Estudio de Validación, Enfermería, Educación en Salud.

## Abstract

**Objectives:**

to develop and validate a model for designing clinical simulation scenarios based on Jeffries Theory.

**Methods:**

qualitative, methodological study based on Pasquali’s psychometrics and Jeffries’ theory, conducted in three phases: (1) theoretical analysis and model development, including expert interviews (n = 20); (2) semantic analysis; and (3) content validation by specialists. Data were analyzed using IRAMUTEQ software. Content Validity Index (CVI) and Cronbach’s alpha were calculated.

**Results:**

a total of 32 professionals participated, mostly nurses with experience in simulation. Theoretical analysis and interviews led to the initial model; semantic analysis refined it; and validation produced the final version. The model showed a 94% CVI and 0.886 Cronbach’s alpha, aligning with Jeffries’ key categories: Context, Background, Design, Simulated Experience, Facilitator, Educational Strategies, Participant, and Outcomes.

**Final Considerations:**

the model was validated, being recommended as a structured and scientifically grounded basis for designing clinical simulation scenarios.

## RESUMEN

**Objetivos:**

construir y validar un modelo metodológico para escenarios de simulación clínica basado en la teoría de Jeffries.

**Métodos:**

estudio cualitativo y metodológico, fundamentado en la psicometría de Pasquali y la teoría de Jeffries, en tres etapas: 1) profundización teórica y construcción del modelo con entrevistas a especialistas (n=20); 2) análisis semántico; 3) validación de contenido. Se analizaron los datos con IRAMUTEQ. Se calcularon el Índice de Validez de Contenido (IVC) y el alfa de Cronbach.

**Resultados:**

participaron 32 profesionales (mayoría enfermeros con experiencia en simulación clínica). El análisis teórico y las entrevistas generaron la versión inicial; el análisis semántico, la segunda; y la validación, la versión final. Se obtuvo un IVC del 94% y un α=0,886. El modelo abarca Contexto, Antecedentes, Diseño, Experiencia simulada, Facilitador, Estrategias educativas, Participante y Resultados.

**Conclusiones:**

el modelo fue validado y se recomienda para diseñar escenarios de simulación clínica como base orientadora y científica.

## INTRODUCTION

Clinical simulation (CS) scenarios are replications of real-world settings, requiring clear validation and reliability processes to ensure their rigorous development^(1,2)^. The expanding use of CS, particularly in nursing, results from methodological advancements strengthened by technological progress^(3)^. When structured and theoretically grounded, CS enables participants to develop competencies in a safe environment where errors can occur without posing risks to patients^(4)^. This method has gained increasing importance in healthcare education and professional training, especially in nursing education^(1,2)^.

The rapid increase in CS centers and training courses using this method-often without rigorous quality assurance or verification of expected outcomes-has driven research to develop validated and reliable simulation scenarios^(2,4,5)^. Although some guidelines and checklists exist for scenario development, the literature highlights a lack of standardization in structural and conceptual design, as well as the frequent omission of scientific and methodological rigor in published studies on scenario development^(2,6,7)^.

Non-validated scenarios lacking scientific grounding can compromise participant performance and assessment, limiting both competency development through simulation and the reproducibility of scenarios across different contexts^(2,6)^. Therefore, ensuring methodological reliability and standardization in scenario development is essential, guaranteeing that the content enables competency development and aligns with participants’ knowledge levels^(6,8)^.

The standardization and theoretical grounding of CS scenario development facilitate the integration of new facilitators into the field, as many avoid the method due to its perceived complexity^(9)^. The theory developed by nurse Pamela Jeffries served as the theoretical framework for this study, given her global recognition in leading research on CS, technological innovation, and health education. Jeffries conceptualizes CS as a method that recreates real clinical environments, enabling the execution of scenarios involving procedures, decision-making, and critical thinking, primarily developed using techniques and technologies such as human patient simulators, interactive videos, and role-playing^(10)^.

Pamela Jeffries’ most prominent contribution was the development of a framework that has guided the implementation and design of CS in healthcare over the past 20 years^(7)^. The evolution of this framework led to Jeffries Simulation Theory, which has undergone multiple revisions to reach its current form. This theory serves as a robust foundation for CS, structured around eight conceptual categories: Context, Background, Design, Simulation Experience, Facilitator, Educational Strategies, Participant, and Outcomes.

The interrelation among these categories defines the fundamental principles for implementing the method, fostering advances in CS practice and research^(7)^. Considering the theory and the lack of standardized, theory-based models for scenario design in the literature, the following research question arises: Which components are suitable for proposing a methodological model for scenario design based on the NLN Jeffries Simulation Theory?

## OBJECTIVES

To develop and validate a methodological model for designing clinical simulation scenarios based on the NLN Jeffries Simulation Theory.

## METHODS

### Ethical aspects

We conducted the study in accordance with national ethical guidelines. The Federal University of Paraná’s Research Ethics Committee approved the study. Informed Consent was obtained electronically from all individuals involved in the study.

### Theoretical-methodological framework

The development of the methodological model followed the theoretical framework of Jeffries Simulation Theory^(7)^ and the methodological framework of Pasquali’s psychometrics^(11)^, a science recommended for designing valid and reliable measurement instruments and scales. Psychometric theory is based on instruments that, to be valid and reliable, must possess essential characteristics related to item analysis and validity. These characteristics, known as *psychometric properties*, were considered in this study.

Additionally, we followed the theoretical and experimental phases of psychometrics, as described in the methodological procedures section.

### Study type

This is a qualitative, methodological, descriptive, and exploratory study conducted between 2020 and 2024. It was guided by the EQUATOR (Enhancing the Quality and Transparency of Health Research) network guidelines for instrument development studies^(12)^. For the qualitative phase, the Consolidated Criteria for Reporting Qualitative Research (COREQ) instrument was used^(13)^.

### Methodological procedures

In the first phase, we conducted a review of Jeffries Simulation Theory, following the theoretical stages of Pasquali’s psychometrics: defining the primary object, selecting its attributes, describing the internal structure of these attributes, and establishing constitutive definitions of the object, i.e., the properties of the construct. Next, individual interviews with experts were conducted as a source for developing the model’s items. This phase resulted in the first version of the model.

In the second phase, we performed a semantic analysis with two population groups-those with less and more experience in clinical simulation. After review and adjustments, the second version of the model was obtained. In the third phase, content validation was carried out, during which the model underwent individual evaluation by expert reviewers, as recommended by Pasquali^(11)^. This final phase produced the definitive version of the model, which is available in the “Data and Material Availability” section.

### Study setting

We conducted all research remotely with healthcare professionals in the field of CS. The first phase involved nurses with at least one year of experience in the field. The second phase included one group of nurses with less experience and another with more experience in CS. The third phase consisted of professionals with over one year of experience and knowledge of Jeffries Simulation Theory.

### Data source

In the first phase, we sent 200 invitations via email to healthcare professionals identified through the Lattes Platform, using the search term “clinical simulation” combined with the snowball sampling technique and searches for authors of articles published in the last five years. The inclusion criteria for participants were: at least one year of experience in CS; or a specialization, master’s, or doctoral degree in the field.

Participant selection criteria followed the methodological framework recommendations, prioritizing experience and/or academic background related to the study’s focus. Since the methodological framework does not specify a minimum number of participants, selection was based on convenience sampling, considering the characteristics of the target population. Ultimately, 20 professionals agreed to participate in the study.

The second phase involved two groups of evaluators, all of whom were nurses. The first group consisted of three nurses with less than one year of experience, while the second included three nurses with more than one year of experience. Including professionals with different levels of experience aimed to verify whether both groups easily understood the instrument.

In the third phase, we selected six nurses based on the following inclusion criteria: at least one year of experience in CS and familiarity with Jeffries Simulation Theory (having published at least one article referencing the author). The sample selection and participant numbers for the second and third phases followed Pasquali’s recommendations^(11)^ and were carried out through convenience sampling via email.

### Data collection and organization

In the first phase, participants completed a sociodemographic questionnaire and took part in individual interviews, which we recorded in audio and video via Microsoft Teams. The interviews included five structured questions, previously tested in a pilot interview with a professional equivalent to the study sample: 1) Which phases do you consider essential for planning and designing clinical simulation scenarios, and why? 2) What characteristics are essential for executing the scenario and achieving the expected outcomes? 3) What factors do you consider limiting or challenging when designing and implementing simulated scenarios in practice? 4) Do you currently use any specific method or theoretical framework in your practice? If so, which one? 5) In your opinion, for a scenario to be considered valid, how important is prior expert review?

The principal author conducted the interviews with an average duration of 30 minutes in the first semester of 2023. She was a nurse and doctoral student then, trained in qualitative research techniques. The purpose was to identify the essential components for the model’s composition. The number of interviewees was sufficient to reach data saturation. There were no dropouts or repeated interviews.

We conducted the second phase via Google Forms, where both groups provided feedback on their understanding of all items in the first version of the model. In the third phase, which focused on content validation, reviewers analyzed the entire model, including theoretical guidelines and the eight categories individually, also via Google Forms.

They rated the items using a four-point Likert scale (not relevant, slightly relevant, relevant, highly relevant) designed by the authors. The evaluation included three criteria: 1) clarity and objectivity - whether the content and items are clearly, precisely, and objectively written; 2) pertinence - whether the content and items are pertinent to the analyzed attribute; and 3) relevance - whether the content and items are relevant in relation to the analyzed attribute.

### Data analysis

The qualitative data collected during the first-phase interviews were transcribed, and content analysis was conducted using the IRAMUTEQ software (Interface de R pour les Analyses Multidimensionnelles de Textes et de Questionnaires), as described by Viegas and Boralis^(14)^. We filtered the database to remove unrecognized terms or those that could distort the results, such as linguistic artifacts.

We organized and categorized the data based on the five formulated questions, generating five distinct, coded text files (from “Interviewee 1” to “Interviewee 20” and “Question 1” to “Question 5”). Depending on the question, we applied various content analysis methods using IRAMUTEQ, including classical textual statistics, Descending Hierarchical Classification (DHC), similarity analysis, and/or word cloud generation. No transcript validation or participant feedback was requested for reanalysis.

In the second phase, we analyzed participants’ understanding of the described items based on content to verify whether they represented the intended construct. Items were adjusted according to interpretation. In the third phase, using the collected data, the Content Validity Index (CVI) was calculated, with a minimum acceptance threshold of 80%, as recommended by the methodological framework^(11)^.

As a measure of reliability, we calculated the overall Cronbach’s alpha (α) coefficient to assess internal consistency across the eight categories. The calculation was performed using the R software for statistical computing, version 4.3.0, with the psych package and a minimum acceptable value of 0.70^(15)^. After analysis, relevant qualitative suggestions from this phase were incorporated into the instrument, resulting in the final version of the model.

## RESULTS

The results from the first methodological phase highlight that the primary object of the model was defined as the CS scenario, and its properties or attributes align with the eight categories of Jeffries Simulation Theory. Through a critical review and analysis of the theory, the internal structure and constitutive definitions of each model attribute were determined, as described in Chart 1.

**Chart 1:** Identified terms related to the internal structure of the methodological model for designing simulation scenarios based on Jeffries Theory

Category	Internal structure terms of the model
**Context**	Location, simulation purpose, circumstances, and environment
***Background***	Expectations, theoretical perspective, and how simulation fits into the curriculum
**Design**	Scenario complexity, realism, fidelity, authenticity, objectives, participant and observer roles, use of audiovisual resources for recording, activity progression, problem-solving and student support, briefing and debriefing strategies, and relationships between design elements and outcomes
**Simulation Experience**	Experiential, interactive, collaborative, learner-centered environment with a sense of psychological safety for all participants
**Facilitator**	Personality, competence, interpersonal relationships, technological skills, attitude, roles, responsibilities, values, self-awareness, teaching ability, and experience
**Educational Strategies**	Facilitator/participant collaboration, interactivity, learner-centered practice, mastery learning, defined outcomes, difficulty level, clinical variation capture, individualized learning, deliberate practice, dose-response, activity sequencing, prebriefing to debriefing, feedback, cues, theory-based practices, curriculum integration, diverse learning approaches, and repeated exposure
**Participant**	Program, educational level, age, gender, readiness to learn, personal objectives, simulation preparation, tolerance for ambiguity, self-confidence, learning style, cognitive load, anxiety level, observer function, and active participant role
**Outcomes**	Learning (knowledge), skill performance, student satisfaction, critical thinking, self-confidence, self-efficacy, behavioral change, patient outcomes, self-awareness, attitudes and empathy, learning transfer, patient safety, cost-effectiveness, and psychological stress of participants

After defining the concepts, we conducted expert interviews (n = 20) as the final phase of the first stage. The participants were predominantly female (80%, n = 16) and most were between 30 and 49 years old (60%, n = 12) or 50 and 65 years old (40%, n = 8). All had a master’s degree as their highest level of education (100%, n = 20), and the majority also held a doctoral degree (60%, n = 12). All participants had more than two years of experience in CS (100%, n = 20), with nearly half having over five years of experience (40%, n = 8). Most were nurses (75%, n = 15), followed by physicians (20%, n = 4) and one physiotherapist (5%, n = 1).

**Figure 1:**
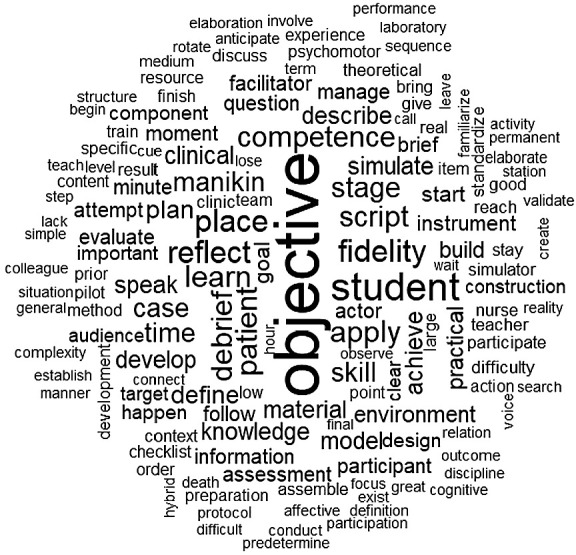
Word cloud analysis of Question 1

Classical textual statistics from Question 1 (analyzed using IRAMUTEQ software) highlighted the most frequently recurring terms: “objective” (78 times), “need” (35), “want” (32), and “think” (31). Word cloud and similarity analyses identified additional relevant terms, such as “student,” “fidelity,” “thinking,” “learning,” “competence,” “debriefing,” “script,” and “use” (Figure 1).

The Descending Hierarchical Classification (DHC) generated four classes: Learning objectives of the scenario necessary for participant development; Material and human resources required for scenario design; Scenario planning and outcomes; and Model, method, and checklist for scenario development and monitoring. The results from this question’s analysis were essential in identifying the key concepts that should be included in the model.

In the analysis of Question 2, two active verbs appeared more than 30 times: “to be” (46 times) and “to think” (33 times). Other terms identified as essential for scenario implementation included “important,” “good,” “need,” “want,” and “realism.” The word “to be” was the most frequently mentioned, often referring to “being present” and “being prepared.”

The word cloud analysis highlighted terms such as “knowledge,” “environment,” “plan,” “planning,” “structure,” “resource,” “important,” “realism,” and “example”. Participants emphasized the importance of methodological knowledge and environment in achieving a realistic CS. Planning was identified as a crucial phase, with Interviewee 1 attributing it to 80% of the simulation process. Additionally, structure and adequate resources were mentioned as contributing factors, although they were not considered the most critical elements.

The DHC analysis of Question 2 generated four distinct classes: Class 1 - The importance of reflection in scenario execution; Class 2 - Knowledge and skills required for scenario execution; Class 3 - The importance of planning in scenario execution; and Class 4 - Structure and resources necessary for effective clinical simulation scenarios. We considered all these concepts during the development of the methodological model.

**Figure 2:**
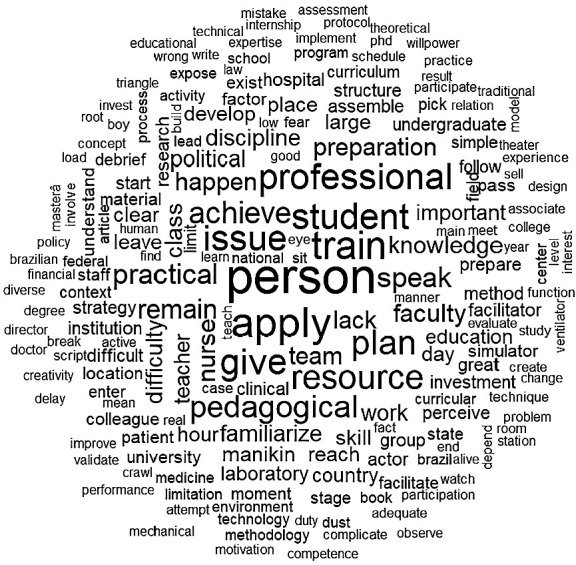
Word cloud analysis of Question 3

The classical textual statistics analysis of Question 3 highlighted the prevalence of terms such as “person” and “time.” Word cloud and similarity analyses emphasized words such as “apply,” “give,” “professional,” “want,” “issue,” “resource,” “course,” and “pedagogical.” These terms are related to the practical execution of clinical simulation, underscoring the importance of professional motivation, practical challenges, and pedagogical and institutional barriers (Figure 2).

Based on the results of this question, the model was designed to help mitigate the limiting factors that hinder the use of clinical simulation in practice. To achieve this, accessible language was adopted, making the model more user-friendly, particularly for professionals with little or no experience in clinical simulation.

The statistical analysis of Question 4 revealed that the most frequently cited expression was “script.” In the word cloud analysis, the most prominent terms were “follow,” “material,” and “Jeffries.” The terms “follow” and “material” were related to adhering to a reference framework or using one’s own instructional material, while “Jeffries” referred to Pamela Jeffries, the theorist on whom the proposed methodological model is based.

Quantitatively, six professionals mentioned Pamela Jeffries as a reference, while three cited Fabri and colleagues^(16)^, another three followed the INACLS recommendations^(17)^, and eight used an instrument developed either by themselves or by their institution. Additionally, three professionals reported not using any specific model. These findings reinforced the need to develop the model proposed in this study.

For Question 5, textual analysis highlighted words such as “specialist,” “validate,” and “example.” Some professionals emphasized the importance of including specialists in CS, while others stressed the relevance of experts in the specific content of the scenario. Regarding the participation of the target audience in the validation process, some argued that repeated scenario application and audience feedback contribute to practical validation, allowing for adjustments as needed. The word cloud analysis identified terms such as “specialist,” “think,” “understand,” “field,” and “speak,” while similarity analysis highlighted “professional,” “student,” and “validate.”

From the first phase, which involved theoretical review and expert consultation, the first version of the model was developed, consisting of 8 categories and 30 items. In the second phase, focused on semantic analysis, two groups of evaluators assessed whether the model’s items were easily understood. All evaluators were nurses with at least a master’s degree as their highest level of education (100%; n = 6). Half of the evaluators had less than one year of experience in clinical simulation (50%; n = 3), while the other half had more than one year of experience. The suggestions provided during this phase resulted in textual adjustments for interpretation in 15 of the 30 model items.

In the third phase, concerning content validation (n = 6), four specialists were female (67%) and aged between 30 and 45 years (67%). Two participants held a master’s degree (33%), while four had a doctoral degree (67%). Additionally, four specialists had over five years of experience (67%). The agreement among evaluators, measured using the Content Validity Index (CVI), was calculated across the eight categories, with a minimum agreement level of 83%. Five categories achieved 100% agreement across all criteria.

In the context, facilitator, and participant categories, an 83% agreement was observed for Criterion 1 (clarity and objectivity). Meanwhile, the facilitator, educational strategies, and participant categories reached 83% agreement for Criteria 2 (pertinence) and 3 (relevance), as presented in Table 1. The overall CVI resulted in a 94% agreement rate, confirming the content validity of the model. We analyzed the experts’ comments and suggestions, leading to modifications that improved clarity and refinement of the model.

**Table 1:** Content Validity Index for each category of the complete model based on the criteria of clarity and objectivity, pertinence, and relevance

Category	CVI		
	Clarity and objectivity %	Pertinence %	Relevance %
Context	83	100	100
Background	100	100	100
Design	100	100	100
Simulation Experience	100	100	100
Facilitator	83	83	83
Educational Strategies	100	83	83
Participant	83	83	83
Outcomes	100	100	100
Complete model	100	100	100
Mean CVI	94	94	94

*CVI - Content Validity Index.*

The Cronbach’s alpha coefficient was calculated for the model as a whole, correlating the eight categories. The final result was 0.886, which is considered excellent. After the phases of development, analysis, and validation, the definitive version of the model was established for scenario design based on Jeffries Simulation Theory. This final version is available in the “Data and Material Availability” section of this article and at the following link: https://doi.org/10.48331/scielodata.QTVEUI.

The model provides theoretical guidelines that conceptually support scenario development and guide the completion of the 30 model items. These items must be completed during the planning and implementation stages of simulation scenarios. They are distributed across the eight categories outlined in the theoretical framework: Context, Background, Design, Simulation Experience, Facilitator, Educational Strategies, Participant, and Outcomes. These categories serve as the foundation for decision-making and facilitate the achievement of successful simulation outcomes.

## DISCUSSION

The development and validation of the model presented in this study were conducted with the theoretical and methodological rigor necessary to create a reliable instrument for guiding the planning and implementation of simulation scenarios. This rigor is essential to ensure competency development, allowing scenarios to dynamically reflect reality and remain relevant across various levels of complexity^(18)^. As demonstrated in this study, the scientific evidence underlying the planning and implementation of these scenarios ensures their validity, usability, and replicability-key characteristics for their adoption in diverse environments and contexts^(19)^.

Based on the eight categories of Jeffries Simulation Theory, the theoretical structure of the model proved effective in integrating fundamental concepts into CS practice^(7,20)^. Regarding the Context category, the model highlighted curricular integration throughout training and the context in which CS is applied, enabling the use of scenarios at different levels of complexity. Contextualizing the scenario-by considering its setting, specific conditions, and the broader simulation environment-is crucial during scenario design to ensure relevance and effectiveness in professional training^(21,22)^.

For the Background category, the model emphasized the importance of the target audience, participants’ prior training, and the scenario’s theoretical framework. Adequate preparation of participants is essential to align knowledge and expectations, which, according to the literature, enhances learning retention and competency development in CS^(23,24)^. These findings reinforce the need for robust preparation before implementing scenarios, thereby ensuring participants are well-equipped to handle simulated situations.

The Design category shows the relevance of clearly defined objectives to ensure an effective simulation experience. The findings of this study and existing literature indicate that well defined objectives not only guide activities but also play a fundamental role in achieving expected outcomes^(25,26)^. Additionally, components such as briefing, debriefing, participant roles, feedback, realism, and scenario fidelity were incorporated into the model, aligning with the relevance of these elements in recent studies^(27,28)^. Therefore, these components contribute to enhancing scenario realism and achieving effective learning outcomes.

Debriefing, in particular, was highlighted in this study as a tool for stimulating critical reflection among participants, ensuring that practice is analyzed, refined, and immediately discussed after scenario execution^(29,30)^. Studies suggest that facilitators should be properly trained in conducting debriefing, as their role is to guide discussions while allowing participants to reach conclusions about their own performance. Feedback, on the other hand, serves as a means to correct potential errors. In this model, it is recommended that feedback be delivered as cues throughout the scenario, as some studies suggest that corrective feedback during debriefing may inhibit reflection and self-criticism^(31)^.

The transition to the Simulation Experience category highlights the dynamic interaction between facilitator and participant, a collaborative learner-centered environment, and the experiential nature of the simulated scenario. These elements are recognized as key factors in competency development, reinforcing the fundamental principles of clinical simulation within the proposed model^(26)^.

In the Facilitator category, essential facilitator characteristics were explored as key factors for successful scenario execution. Both the findings of this study and existing literature indicate that the effectiveness of simulation depends not only on technical structure but also on the facilitator’s interpersonal and teaching skills, which are crucial for creating a safe and productive learning environment^(32)^.

Regarding the Educational Strategies category, the model highlighted the use of learner centered techniques, showing that gradual and repeated exposure to scenarios is an effective strategy for developing competencies^(33)^. In the Participant category, aspects such as target audience, level of training, number of participants, and the roles they assume in the scenario were considered. The data support the hypothesis that these approaches not only enhance skills but also contribute to long-term knowledge retention, a crucial factor for clinical practice that should be taken into account when implementing simulation scenarios^(34,35)^.

For the Outcomes category, the expanded focus to include patient-related effects and cost-effectiveness for healthcare systems introduces a new dimension to the discussion. This highlights the benefits of CS beyond participant learning, demonstrating its role in advancing the field^(36,37)^. The correlation between competency development and improved clinical outcomes, as well as cost reduction, suggests that CS can be an effective strategy from both an educational and economic perspective, a conclusion further supported by the literature^(38)^.

During the interview process, additional terms and concepts were incorporated into the methodological model, demonstrating that its development was dynamic and adaptable to practical needs. In addition to clearly defining objectives, interviewees reiterated that planning is a fundamental phase, confirming the hypothesis that effective planning is critical to the success of the model. The findings of this study align with the literature, which highlights planning-one of the most discussed aspects of this model-as essential in scenario design, ensuring the necessary conditions for successful implementation^(39)^.

The emphasis on identifying material and human resources, as well as the standardization of scenario design methods using checklists and evaluation tools, underscores these elements as crucial in the planning process, which was reflected in the model. Although some resources may be expensive, the literature indicates that they are not the determining factor for successful outcomes^(40)^. This suggests that the effectiveness of the model depends more on standardization and the use of validated tools than on the availability of resources. Using standardized and validated instruments enables reliable assessment of CS characteristics, including skills, competencies, perceptions, and participant performance, among other aspects^(39,41,42)^.

Interviewees emphasized the importance of physical infrastructure and available resources. The literature suggests that well-equipped CS laboratories with modern resources provide advantages, particularly in terms of scenario variability^(43)^. Although laboratory quality may vary, the data from this study indicate that infrastructure is an important but non-essential element for CS success. For example, a study conducted in India found that only 44% of the laboratories evaluated had an adequate structure for simulations^(44)^. These findings reveal that considering these aspects in the model design is essential.

The knowledge and skills of those involved in scenario execution were strongly emphasized by the interviewees, highlighting that facilitator training is a key factor in ensuring the effectiveness of CS. The lack of trained professionals remains an obstacle to the widespread adoption of CS. Studies indicate that while many educators express a positive attitude toward CS, they often face challenges related to technological use and facilitation skills^(45)^. This finding reinforces the urgent need to invest in the training of these professionals.

When asked about barriers to using CS, interviewees cited challenges related to professional attitudes, course curriculum design, facilitator training, and the availability of resources and time. These results suggest that CS requires a proactive approach and a shift in mindset regarding traditional teaching practices. Therefore, aligning CS with educational curricula is essential for integrating it into training programs, overcoming time constraints, and ensuring a structured approach to its implementation, as supported by the literature^(22)^.

Facilitator training and qualification emerged as a key factor, reinforcing previous studies that show the urgent need for investments in the education and professional development of those involved in CS^(45)^. Financial constraints, lack of technological infrastructure, and time limitations were identified as perceived barriers, highlighting the need to overcome logistical and bureaucratic challenges to enable the implementation of high-quality CS practices^(9)^.

The results of this study indicate that most interviewees use their own scenario script, emphasizing the importance of this methodological model in providing scientific rigor and guidance for facilitators during scenario execution. The primary hypothesis suggests that the lack of validated scripts based on a theoretical framework represents a weakness in CS development, as reinforced by Nascimento et al.^(46)^. Furthermore, Jeffries was cited as the most widely used reference, confirming the significant influence of her theoretical framework in this field.

Scenario validation by experts is recognized as a critical step in CS development, as it ensures reliability, alignment with learning objectives, and scenario refinement^(47)^. The interviewees highlighted the need for experts in the scenario’s subject matter, ensuring scenarios are tailored to participants’ realities and grounded in scientific evidence^(48)^.

Semantic analysis aimed to assess whether the model’s items were understandable for participants with different levels of experience, while content validation sought to ensure that the content was appropriate and accurately represented the intended learning objectives. The expertise and qualifications of the evaluators were highlighted as key factors contributing to the quality and reliability of content validation results^(19)^.

An analysis of the results indicates that the developed methodological model meets the criteria of clarity and objectivity, pertinence, and relevance, as validated by expert judges with an agreement rate exceeding 80%, reinforcing its robustness. Comparisons with similar studies, such as checklists and CS scenarios, along with an in-depth discussion of the categories, further support the model’s validity^(42,49,50)^. The positive results, with a global CVI of 94% and a general Cronbach’s alpha coefficient of 0.886, indicate high reliability and strong agreement among experts. The absence of similar models in the literature highlights this model’s uniqueness and innovative nature, which not only addresses an existing gap but also establishes new guidelines for practice.

The findings of this study reveal that meticulous planning, training of involved professionals, and the standardization of resources and processes are critical factors for the success of CS. Curricular integration, overcoming logistical barriers, and continuous professional development are essential to maximize the positive impact of CS in healthcare education, ensuring not only educational effectiveness but also continuous improvement in patient care.

### Study limitations

A limitation of this study is the relatively recent recognition of Jeffries Simulation Theory within the CS landscape despite the widespread acknowledgment of her framework. Additionally, we suggest validating the instrument with a larger sample than the one used in this study, which followed content analysis recommendations for instrument development. It is also important for future validations to include more robust statistical data.

### Contributions to the field of health and nursing

This study aims to contribute to the scientific rigor in the development and implementation of simulation scenarios and to facilitate the dissemination of CS, thereby supporting healthcare education, patient safety, quality improvement in care delivery, and the advancement of knowledge in the field of nursing.

## FINAL CONSIDERATIONS

This study achieved its objective of developing and validating a methodological model for scenario design based on Jeffries Simulation Theory and Pasquali’s rigorous psychometric approach, making it suitable for sharing with CS professionals. By making this model available to the academic community, we aim to enhance scientific rigor in developing and implementing simulation scenarios, particularly in nursing.

Additionally, we expect this model to facilitate its adoption among educators and facilitators with little or no experience in CS, as well as among those who may be hesitant to use the method. As a result, this model may standardize scenario design, enabling broad application across different contexts and allowing for comparative studies.

Providing this methodological model for CS scenario design offers a structured, evidence based framework for educators and facilitators. The goal is to ensure that the method is effectively applied, contributing to the competency development of students and professionals, patient safety, and improving the quality of care.

## Data Availability

*https://doi.org/10.48331/scielodata.QTVEUI*

## References

[1] Kaneko RMU, Lopes MHB. (2019). Realistic health care simulation scenario: what is relevant for its design?. Rev Esc Enferm USP.

[2] Mirza N, Cinel J, Noyes H, Mckenzie W, Burgess K, Blackstock S (2019). Simulated patient scenario development: a methodological review of validity and reliability reporting. Nurse Educ Today.

[3] Martins JCA, Mazzo A, Baptista RCN, Coutinho VRD, Godoy S, Mendes IAD (2020). The simulated clinical experience in nursing education: a historical review. Acta Paul Enferm.

[4] Assis MS, Nascimento JSG, Nascimento KG, Torres GAS, Pedersoli CE, Dalri MCB. (2021). Simulation in nursing: production of the knowledge of the graduate courses in Brazil from 2011 to 2020. Texto Contexto Enferm.

[5] Cazañas EF, Prado RL, Nascimento TF, Tonhom SFR, Marin MJS. (2021). Simulation in nursing baccalaureate courses of Brazilian educational institutions. Rev Bras Enferm.

[6] Almeida AOD, Dantas SRPE, Paula MAB, Silva JLG, Franck EM, Oliveira-Kumakura ARS (2021). Development, validation and application of clinical simulation scenarios for assessment of stomatherapy specialists. Rev Bras Enferm.

[7] Jeffries PR. (2022). The NLN Jeffries Simulation Theory.

[8] Associação Brasileira de Educação Médica (ABEM) (2021). Simulação em Saúde para ensino e avaliação: conceitos e práticas.

[9] Bahia BM, Souza MG, Jaqueira RSP, Buchidid R, Antonietti CC. (2021). Technical training and practical performance of the teacher in the face of realistic simulation: scope study. REVISA.

[10] Jeffries PR. (2012). Simulation in Nursing education: from conceptualization to evaluation.

[11] Pasquali L. (2013). Psicometria: teoria dos testes na psicologia e na educação.

[12] Streiner DL, Kottner J. (2014). Recommendations for reporting the results of studies of instrument and scale development and testing. J Adv Nurs.

[13] Souza VRS, Marziale MHP, Silva GTR, Nascimento PL. (2021). Translation and validation into Brazilian Portuguese and assessment of the COREQ checklist. Acta Paul Enferm.

[14] Viegas RR, Borali N. (2022). Análise de conteúdo e o uso do Iramuteq. Rev Lat Am Metodol Investig Soc.

[15] Hair JF, Black WC, Babin BJ, Anderson RE, Tatham RL. (2009). Análise multivariada de dados.

[16] Fabri RP, Mazzo A, Martins JCA, Fonseca AS, Pedersoli CE, Miranda FBG (2017). Development of a theoretical-practical script for clinical simulation. Rev Esc Enferm USP.

[17] INACSL Standards Committee (2021). Healthcare simulation standards of best practice simulation design. Clin Simul Nurs.

[18] Lima LG, Draganov PB, Sampietri IC, Saito KAM, Balsanelli AP. (2023). Construction and validation of a clinical simulation scenario for teaching conflict management. Cogitare Enferm.

[19] Barboza ES, Almeida RGS, Girão FB, Negri EC, Ferreira Júnior, MA, Jorge BM (2023). Construction and validity of scripts for skills training on enteral nutritional therapy in dehospitalization. Texto Contexto Enferm.

[20] Jeffries PR, Rodgers B, Adamson K. NLN (2015). Jeffries Simulation Theory: brief narrative description. Nurs Educ Perspect.

[21] Herrera-Aliaga E, Estrada LD. (2022). Trends and Innovations of Simulation for Twenty First Century Medical Education. Front Public Health.

[22] Ayaz O, Ismail FW. (2022). Healthcare Simulation: a key to the future of medical education, a review. Adv Med Educ Pract.

[23] Tiu RA, Meyer TK, Mayerhoff RM, Ray JC, Kritek PA, Merati AL (2022). Tracheotomy care simulation training program for inpatientproviders. Laryngoscope Investig Otolaryngol.

[24] Mukhtar K, Javed K, Arooj M, Sethi A. (2020). Advantages, Limitations and Recommendations for online learning during COVID-19 pandemic era. Park J Med Sci.

[25] Nunes JGP, Freitas P, Bergamasco EC, Cruz DALM. (2022). Implementation of good practices in clinical simulation in nursing education. Acta Paul Enferm.

[26] Silva SR, Diniz SN. (2023). Construção do cenário em simulação clínica. Hum Tecnol.

[27] Mandelbaum MHS, Martins TMS, Porfirio R, Melaragno ALP, Visentin A, Francisconi R., Melaragno ALP (2023). Glossário Educação em Saúde. Educação Permanente em Saúde.

[28] Moretza-Bagi HR, Ghaffarzad A, Fathipour P, Yazdani R, Khamnian Z, Rahnemayan S. (2022). The effect of teacher-made simulation moulage on learning cricothyrotomy skills in emergency medicine physicians. J Emerg Pract Trauma.

[29] Cust F, Boden R. (2022). Exploring the use of pre-briefing and debriefing in educational settings. Clin Pract Disc Nurs Educ.

[30] Fegran L, Ham-Baloyi WT, Fossum M, Hovland OJ, Naidoo JR, Rooyen DV (2023). Simulation debriefing as part of simulation for clinical teaching and learning in nursing education: a scoping review. Nurs Open.

[31] Seo YH, Eom MR. (2021). The effect of simulation nursing education using the outcome-present state-test model on clinical reasoning, the problem-solving process, self-efficacy, and clinical competency in Korean Nursing Students. Healthcare.

[32] Davidson B, Howells S, Davenport R. (2023). “Same But Different”: the role and perceptions of the simulation clinical educator. Teach Learn Commun Sci Disord.

[33] Vitale KM, Barsuk JH, Cohen ER, Wayne DB, Hansen RN, Williams LM (2023). Simulation-based Mastery Learning Improves Critical Care Skills of Advanced Practice Providers. ATS Scholar.

[34] Tutticci N, Theobald KA, Ramsbotham J, Johnston S. (2022). Exploring the observer role and clinical reasoning in simulation: a scoping review. Nurs Educ Pract.

[35] Fonseca AS, Reis F, Melaragno ALP., Melaragno ALP, Fonseca AS, Assoni MAS, Mandelbaum MHS (2023). Habilidades para as melhores práticas clínicas. Educação Permanente em Saúde.

[36] Tavares APM, Rocha DM, Abreu IM, Mendes PM, Avelino FVSD, Barlem JGT. (2022). Instrumentos de medida para avaliação do conhecimento de estudantes de enfermagem sobre segurança do paciente. Enferm Foco.

[37] Santos CA, Siqueira DS, Silva EF. (2023). Segurança do paciente cirúrgico pediátrico: uma revisão integrativa. Espaço Saúde.

[38] Santos ISN, Souza CJ, Silvino ZR, Escudeiro CL, Ferreira RA, Azevedo TT (2023). O uso da simulação clínica na efetivação de competências e habilidades na parada cardíaca para estudantes de enfermagem. Rev Eletrôn Acervo Saúde.

[39] Silva CC, Natarelli TRP, Domingues AN, Fonseca LMM, Melo LL. (2022). Prebriefing in clinical simulation in nursing: scoping review. Rev Gaucha Enferm.

[40] Luebbert R, Perez A, Andrews A, Webster-Cooley T. (2023). Standardized Patients Versus Mannequins in Mental Health Simulation. Sage J.

[41] Das V, Daniels B, Kwan A, Saria V, Das R, Pai M (2023). Simulated patients and their reality: an inquiry into theory and method. Soc Sci Med.

[42] Ravagnani PAL, Oliveira TMM, Rocco KMW, Pereira MGN, Martini JG, Dellaroza MSG (2023). Checklist de competências clínicas no manejo da parada cardiorrespiratória: construção e validação de conteúdo. Rev Contempor.

[43] Shaaban SS, Hassan MS, Mohamed AH. (2021). Comparison between Low and High-Fidelity Simulation regarding Nursing Students’ Self-confidence, Achievement and Satisfaction. Egyptian J Health Care.

[44] Goswami G, Sharma SK, Sharma R, Rani R. (2021). Simulation and Skill Training Facilities in Nursing Institutes at Uttarakhand: a cross sectional study. Iranian J Nurs Midwifery Res.

[45] Akhter Z, Malik G, Plummer V. (2021). Nurse educator knowledge, attitude and skills towards using high-fidelity simulation: a study in the vocational education sector. Nurs Educ Pract.

[46] Nascimento JSG, Nascimento KG, Regino DSG, Alves MG, Oliveira JLG, Dalri MCB. (2021). Clinical simulation: construction and validation of a script for Basic Life Support in adults. Rev Enferm UFSM.

[47] Silva SCN, Alencar BR, Viduedo AFS, Ribeiro LM, Leon CGRMP, Schardosim JM. (2021). Management of severe preeclampsia in the puerperium: development and scenario validation for clinical simulation. Rev Bras Enferm.

[48] Flausino DA, Oliveira AR, Misko MD, Eduardo AHA. (2022). Scenario for simulation training on the communication of hard news: a validation study. Esc Anna Nery.

[49] Dias AA, Costa YCN, Tony ACC, Alvim ALS, Prado RT, Santos KB (2023). Construction and validation of a clinical scenario and checklist for assessing cardiopulmonary resuscitation skills. Cogitare Enferm.

[50] Bernardinelli FBP, Amorim GC, Nascimento JSG, Fonseca LMM, Domingues TAM, Condeles PC (2024). Development of a telesimulation design for basic life support. Acta Paul Enferm.

