# Supplementary material for: Construction and validation of a model for creating simulated scenarios based on Jeffries Theory
Source: Rev Bras Enferm. 2025 Aug 8;78(3):e20240222. doi: 10.1590/0034-7167-2024-0222 (PMC12334201; doi:10.1590/0034-7167-2024-0222)
Supplement: Translation

# Translation: Construção e validação de modelo para elaboração de cenários simulados baseado na teoria de Jeffries

**DOI:** 10.1590/0034-7167-2024-0222pt

**Authors:** Jéssica de Oliveira Veloso Vilarinho, Marcia Bucco, Ana Elizabeth Lopes de Carvalho, Nilton Orlando da Silva, Luciana Puchalski Kalinke, Jorge Vinícius Cestari Felix

**Affiliations:** I Universidade Federal do Paraná. Curitiba, Paraná, Brasil

**Keywords:** Psicometria, Treinamento Simulado, Estudos de Validação, Enfermagem, Educação em Saúde.

## Abstract

**Objetivos:**

construir e validar modelo metodológico para elaborar cenários de simulação clínica baseado na teoria de Jeffries.

**Métodos:**

estudo qualitativo e metodológico, fundamentado na psicometria de Pasquali e teoria de Jeffries, com etapas: 1) aprofundamento teórico e construção do modelo, entrevistando especialistas (n = 20); 2) análise semântica; 3) validação de conteúdo. Os dados de entrevista foram analisados com *software* IRAMUTEQ. Calcularam-se Índice de Validade de Conteúdo (IVC) e alfa de Cronbach.

**Resultados:**

participaram 32 profissionais (maioria enfermeiros com experiência em simulação clínica). Análise teórica e entrevistas geraram versão inicial; análise semântica, a segunda versão; e a validação gerou versão final. Obteve-se alto IVC de 94% e α=0,886. O modelo atendeu às categorias da teoria: Contexto, *Background*, Design, Experiência simulada, Facilitador, Estratégias educacionais, Participante e Resultados.

**Considerações Finais:**

o modelo foi validado, sendo recomendado para construir cenários de simulação clínica como base orientadora e cientificamente fundamentada.

## INTRODUÇÃO

Cenários de simulação clínica (SC) são replicações da realidade e processos claros de validação e confiabilidade, fatores necessários para o seu rigoroso desenvolvimento^(1,2)^. A expansão no uso da SC, especialmente na enfermagem, é também o resultado da evolução do método, que se fortaleceu com o avanço tecnológico^(3)^. Se conduzida de maneira estruturada e teoricamente embasada, a SC propicia ao participante a obtenção de resultados consideráveis no desenvolvimento de competências em ambiente seguro, no qual falhas podem ocorrer sem o risco de danos aos pacientes^(4)^. Esse método tem ganhado cada vez mais relevância na formação e capacitação de profissionais de saúde, notoriamente na formação de enfermeiros^(1,2)^.

O crescente aumento dos centros de SC e dos cursos de capacitação com uso desse método, muitas vezes sem o rigor e garantia do alcance dos resultados esperados, tem motivado estudos de desenvolvimento de cenários válidos e confiáveis^(2,4,5)^. Apesar da existência de alguns roteiros e checklists para a elaboração de cenários, observa-se na literatura a ausência de padrão no design estrutural e conceitual ou até supressão das informações acerca do rigor científico-metodológico nas publicações sobre construção de cenários^(2,6,7)^.

Cenários não validados e sem embasamento científico podem comprometer o desempenho e a avaliação dos participantes e limitar tanto o desenvolvimento de competências com uso do método quanto a reprodutibilidade dos cenários em diferentes contextos^(2,6)^. Dessa forma, é essencial que haja confiabilidade e padronização metodológica na construção de cenários a fim de garantir que o conteúdo expresso permita o desenvolvimento de competências e o desempenho adequado do participante de acordo com seu nível de conhecimento^(6,8)^.

A padronização e a fundamentação na construção de cenários de SC facilitam a adesão de novos facilitadores à área, que muitas vezes evitam o método por considerá-lo complexo^(9)^. A teoria da enfermeira Pamela Jeffries foi utilizada como referencial desta pesquisa, dado o seu reconhecimento mundial na liderança de estudos de SC, inovação tecnológica e pesquisas em educação na área da saúde. Jeffries conceitua a SC como um método que recria ambientes clínicos reais, permitindo a execução de cenários envolvendo procedimentos, tomada de decisão e pensamento crítico, elaborados principalmente mediante técnicas e tecnologias como simuladores humanos, vídeos interativos e encenação de papéis diversos^(10)^.

O projeto de maior destaque de Pamela Jeffries foi o desenvolvimento de um *framework*, que tem guiado a implementação e design da SC na área da saúde nos últimos 20 anos^(7)^. A evolução do *framework* culminou na teoria de simulação de Jeffries, que passou por diferentes revisões até chegar ao formato atual. Essa teoria surge como referencial robusto para utilização da SC, fundamentada e guiada por oito categorias conceituais: Contexto, *Background*, Design, Experiência simulada, Facilitador, Estratégias educacionais, Participante e Resultados.

A inter-relação dessas categorias define as premissas básicas para implementação do método, de forma que promove avanços na prática e pesquisa em SC^(7)^. Considerando a teoria e a ausência de modelos padronizados e embasados em teorias para elaboração de cenários na literatura, surge a seguinte questão de pesquisa: Quais componentes são adequados para a proposição de um modelo metodológico de elaboração de cenários à luz da teoria de Jeffries?

## OBJETIVOS

Construir e validar um modelo metodológico para elaborar cenários de simulação clínica baseado na teoria de simulação de Jeffries.

## MÉTODOS

### Aspectos éticos

O estudo foi conduzido de acordo com as diretrizes de ética nacionais e aprovado pelo Comitê de Ética em Pesquisa da Universidade Federal do Paraná. O Consentimento Livre e Esclarecido foi obtido de todos os indivíduos envolvidos no estudo, por meio eletrônico.

### Referencial teórico-metodológico

A elaboração do modelo metodológico seguiu o referencial teórico da teoria de simulação de Jeffries^(7)^ e referencial metodológico da psicometria de Pasquali^(11)^, ciência recomendada para a construção de instrumentos e escalas de medida válidas e confiáveis. A teoria psicométrica baseia-se em instrumentos que, para serem válidos e confiáveis, devem possuir características essenciais relacionadas à análise dos itens e validade. Tais características são conhecidas como “propriedades psicométricas” e foram consideradas neste estudo. Além disso, foram seguidas as fases teóricas e experimentais da psicometria, descritas na sessão de procedimentos metodológicos.

### Tipo de estudo

Trata-se de um estudo qualitativo, metodológico, descritivo e exploratório, desenvolvido entre 2020 e 2024. Para norteá-lo, foram utilizadas as diretrizes da EQUATOR para estudos de desenvolvimento de instrumentos^(12)^_,_ sendo usado, na etapa qualitativa, o instrumento *Consolidated Criteria for Reporting Qualitative Research* (COREQ)^(13)^.

### Procedimentos metodológicos

Na primeira etapa, foi feita a revisão da teoria de Jeffries e foram seguidas as fases teóricas da psicometria de Pasquali: definição do objeto principal, eleição dos atributos desse objeto, descrição da estrutura interna dos atributos e definições constitutivas do objeto, ou seja, das propriedades do construto. Em seguida, foram realizadas entrevistas individuais com especialistas como fonte para construir os itens. A primeira etapa deu origem à primeira versão do modelo.

Na segunda etapa, procedeu-se à análise semântica para dois grupos de população, com menos e mais experiência em SC. Após revisão e adequação, obteve-se a segunda versão do modelo. Na terceira etapa, foi realizada a validação de conteúdo, na qual o modelo foi submetido ao julgamento individual de avaliadores especialistas, conforme recomenda Pasquali^(11)^. Essa última etapa deu origem à versão final do modelo, disponível na seção “Disponibilidade de dados e material”.

### Cenário do estudo

Toda a pesquisa foi realizada de forma remota com profissionais da saúde da área de SC. A primeira etapa envolveu enfermeiros com ao menos um ano de experiência na área. A segunda abrangeu um grupo de enfermeiros com menos experiência e outro com mais experiência. Já a terceira etapa consistiu em um grupo com mais de um ano de experiência e com conhecimento da teoria de Jeffries.

### Fonte de dados

Na primeira etapa, foram enviados 200 convites por e-mail para profissionais de saúde encontrados na plataforma Lattes mediante o buscador “simulação clínica” em associação à técnica de “bola de neve” (*snowball sampling*) e busca por autores de artigos publicados nos últimos cinco anos. A inclusão dos participantes seguiu estes critérios: possuir experiência mínima de um ano na área de SC; ou especialização, mestrado ou doutorado na área.

Os critérios de seleção dos participantes seguiram as recomendações do referencial metodológico, de experiência e/ou formação sobre o objeto de escolha do estudo. O referencial metodológico não discorre sobre mínimo de participantes necessários, portanto a seleção foi definida por conveniência, considerando as características da população. Por fim, 20 profissionais aceitaram participar da pesquisa.

A segunda etapa foi efetuada por dois grupos de avaliadores, todos enfermeiros. O primeiro foi composto por três enfermeiros com menos de um ano de experiência na área; e o segundo, por três enfermeiros com mais de um ano de experiência. A inclusão de profissionais com diferentes experiências visa confirmar se o instrumento é de fácil entendimento para as duas populações.

Na terceira etapa, foram selecionados seis participantes, todos enfermeiros, preenchendo os seguintes critérios de inclusão: ter mais de um ano de experiência e conhecer a teoria de Jeffries (possuir ao menos uma publicação de artigo em que cite a autora). As seleções e quantidades das amostras da segunda e terceira etapas seguiram as recomendações de Pasquali^(11)^, realizadas por conveniência e por e-mail.

### Coleta e organização dos dados

Na primeira etapa, os profissionais responderam a um questionário sociodemográfico e participaram de entrevistas individuais gravadas em áudio e vídeo, via *Microsoft Teams.* Foram cinco perguntas estruturadas e previamente testadas em uma entrevista-piloto com um profissional equivalente aos da amostra: 1) Quais etapas você considera essencial para o planejamento e construção de cenários em simulação clínica e por quê? 2) Que características são imprescindíveis para a execução do cenário e obtenção dos resultados? 3) Quais fatores você considera limitantes ou dificultadores para que as pessoas construam e executem cenários simulados em suas práticas? 4) Você já utiliza algum método ou referencial na sua prática? Se sim, qual? 5) Para que o cenário seja considerado válido, qual a sua opinião sobre a análise prévia do cenário por especialistas?

Com média de 30 minutos, as entrevistas foram realizadas no primeiro semestre de 2023 e conduzidas pela autora principal, que é enfermeira, doutoranda à época e com capacitação para uso da técnica. O propósito foi identificar quais componentes eram essenciais na composição do modelo. O número de entrevistados foi suficiente para haver saturação dos dados. Não houve desistência dos entrevistados nem repetição de entrevistas.

A segunda etapa foi realizada via Google Forms, na qual as duas populações puderam descrever o entendimento de todos os itens da primeira versão do modelo. Na terceira etapa, de validação do conteúdo, os avaliadores puderam analisar o conteúdo do modelo completo (orientações teóricas e as oito categorias de forma individual) via Google Forms.

Eles preencheram uma escala do tipo Likert de quatro pontos (não pertinente, pouco pertinente, pertinente, muito pertinente), confeccionada pelos autores, que incluía análise sobre 1) clareza e objetividade - se o conteúdo e o item têm redação clara, precisa e objetiva; 2) pertinência - se o conteúdo e o item são pertinentes ao atributo analisado; e 3) relevância - se o conteúdo e o item são relevantes em relação ao atributo analisado.

### Análise dos dados

Os dados qualitativos coletados nas entrevistas da primeira etapa foram transcritos, e foi feita a análise do conteúdo com uso do *software* IRAMUTEQ (*Interface de R pour les Analyses Multidimensionnelles de Textes et de Questionnaires*), conforme descrito por Viegas e Boralis^(14)^. A base de dados foi filtrada, removendo termos não reconhecidos pelo *software* ou que poderiam distorcer os resultados, como vícios de linguagem.

Os dados foram organizados e divididos de acordo com as cinco perguntas formuladas, gerando cinco arquivos de texto distintos e codificados (do “Entrevistado 1” ao “Entrevistado 20” e da “Pergunta 1” à “Pergunta 5”). A depender da pergunta, foram aplicados métodos de análise de conteúdo com IRAMUTEQ como estatísticas textuais clássicas, Classificação Hierárquica Descendente (CHD), análise de similitude e/ou nuvem de palavras. Não houve devolução da transcrição ou feedback aos participantes para reanálise.

Na segunda etapa, o entendimento dos itens descritos pelos participantes foi analisado levando-se em conta o conteúdo, para verificar se representava o construto pretendido. De acordo com a interpretação, o item foi ajustado. Na terceira etapa, com os dados coletados, foi calculado o Índice de Validade de Conteúdo (IVC), aceitando o mínimo de 80% de acordo com o referencial^(11)^.

Como medida de confiabilidade, foi calculado o coeficiente alfa (α) de Cronbach geral do modelo, com teste de consistência interna correlacionado às oito categoriais. No cálculo desse coeficiente, foi utilizado o *software* R para computação estatística, pacote psych, versão 4.3.0, com valor mínimo aceitável de 0,70^(15)^. Após análise, as sugestões qualitativas pertinentes a essa etapa foram incluídas no instrumento, resultando na versão final do modelo.

## RESULTADOS

Os resultados da primeira etapa metodológica destacam que o objeto principal do modelo foi definido como o cenário de SC, e as propriedades ou atributos do modelo consistem nas oito categorias da teoria de Jeffries. Na revisão e leitura crítica da teoria, foi determinada a estrutura interna e as definições constitutivas de cada atributo do modelo, descritas no Quadro 1.

**Quadro 1:** Achados de termos da estrutura interna do modelo metodológico para construção de cenários à luz da teoria de Jeffries

Categoria	Termos da estrutura interna do modelo
**Contexto**	Local, propósito da simulação, circunstância e ambiente
***Background***	Expectativas, perspectiva teórica e como a simulação se encaixa no currículo
**Design**	Complexidade do cenário, realismo, fidelidade, autenticidade, objetivos, papéis do participante e do observador, uso de recursos audiovisuais para gravação, progressão das atividades, resolução de problemas e suporte ao estudante, estratégias de *briefing* e *debriefing* e relações entre recursos do design e resultados
**Experiência simulada**	Ambiente experiencial, interativo, colaborativo, centrado no aluno e com confiança para todos
**Facilitador**	Personalidade, competência, relações interpessoais, habilidades tecnológicas, atitude, papéis, responsabilidades, valores, autoconsciência, capacidade de ensino e experiência
**Estratégias educacionais**	Colaboração facilitador/participante, interatividade, prática centrada no aprendiz, aprendizagem de domínio, resultados definidos, nível de dificuldade, captura de variação clínica, aprendizagem individualizada, prática deliberada, dose-resposta, sequenciamento de atividades, pré-*briefing* até *debriefing,* feedback, dicas, práticas baseadas em teoria, integração curricular, aprendizagem diversificada e exposição repetida
**Participante**	Programa, nível de formação, idade, gênero, prontidão para aprender, objetivos pessoais, preparação para simulação, tolerância para ambiguidade, autoconfiança, estilo de aprendizagem, carga cognitiva, nível de ansiedade, função de observador e papel de participante ativo
**Resultados**	Aprendizado (conhecimento), desempenho da habilidade, satisfação do aluno, pensamento crítico, autoconfiança, autoeficácia, mudança de comportamento, resultados do paciente, autoconsciência, atitudes e empatia, transferência de aprendizagem, segurança do paciente, custo-efetividade e estresse psicológico dos participantes

Após definidos os conceitos, procedeu-se à entrevista com especialistas (n = 20), última fase da primeira etapa. Os participantes eram majoritariamente do sexo feminino (80%, n = 16), com idade prevalente entre 30 a 49 anos (60%; n = 12) e 50 a 65 anos (40%; n = 8). Todos tinham o mestrado como última formação (100%, n = 20) e a maioria também o doutorado (60%, n = 12). Todos possuíam experiência com a SC acima de dois anos (100%, n = 20), com quase a metade acima de cinco anos (40%, n = 8). Em sua maioria, eram enfermeiros (75%, n = 15), e os demais eram médicos (20%, n = 4) e fisioterapeuta (5%, n = 1).

A estatística textual clássica (*software* IRAMUTEQ) da Pergunta 1 destacou os termos com maior recorrência: “objetivo” (78 vezes), “precisar” (35), “querer” (32) e “achar” (31). As análises por nuvem de palavras e de similitude identificaram outros termos relevantes como “aluno”, “fidelidade”, “pensar”, “aprendizagem”, “competência”, “*debriefin*g”, “roteiro” e “utilizar” (Figura 1).

**Figura 1:** Análise por nuvem de palavras da Pergunta 1

Na Classificação Hierárquica Descendente (CHD), geraram-se quatro classes: Objetivos de aprendizagem do cenário necessários ao desenvolvimento do participante; Recursos materiais e humanos necessários para construção do cenário; Planejamento e desfecho do cenário; e Modelo, método e checklist para construção e acompanhamento do cenário. Os resultados das análises dessa pergunta foram essenciais para identificar quais conceitos deveriam constar no modelo.

Na análise da Pergunta 2, duas formas ativas surgiram mais de 30 vezes: “estar” (46 vezes) e “achar” (33 vezes). Também foram encontrados “importante”, “bom”, “precisar”, “querer” e “realismo” como necessários para a implementação do cenário. A palavra “estar” foi a mais citada, com referência a “estar presente” e “preparado”.

A análise por nuvem de palavras destacou palavras como “conhecimento”, “ambiente”, “planejar”, “planejamento”, “estrutura”, “recurso”, “importante”, “realismo” e “exemplo”. Os participantes ressaltaram a importância do conhecimento metodológico e do ambiente para uma SC realista. O “planejamento” foi enfatizado como etapa essencial, descrito pelo Entrevistado 1 como responsável por 80% da SC. Além disso, a “estrutura” e os “recursos adequados” foram mencionados como aspectos que contribuem, ainda que não sejam os fatores mais importantes.

Na análise CHD da Pergunta 2, foram geradas quatro classes distintas: Classe 1 - A importância da reflexão na execução do cenário; Classe 2 - Conhecimento e habilidades necessárias para execução de cenário; Classe 3 - A importância do planejamento na execução de cenários; Classe 4 - Estrutura e recursos necessários para uma boa execução de cenários de SC. Todos esses conceitos foram considerados durante a elaboração do modelo metodológico.

Na estatística textual clássica da Pergunta 3, evidenciou-se a prevalência de termos como “pessoa” e “vez”. As análises por nuvem de palavras e de similitude ressaltam termos como “aplicar”, “dar”, “profissional”, “querer”, “questão”, “recurso”, “curso” e “pedagógico”. Esses termos ligam-se à execução prática da SC, com destaque para a importância da motivação profissional, dificuldades práticas, bem como obstáculos pedagógicos e institucionais (Figura 2).

**Figura 2:** Análise por nuvem de palavras da Pergunta 3

Com os resultados dessa pergunta, foi possível nortear o modelo para que ele contribuísse para minimizar os fatores limitantes ou dificultadores para uso da SC em suas práticas. Para isso, foi estabelecido o uso de uma linguagem acessível aos interessados da área, especialmente aos profissionais com pouca ou nenhuma experiência em SC.

A análise estatística da Pergunta 4 revelou que a expressão mais citada foi “roteiro”. Na análise por nuvem de palavras, sobressaem-se termos como “seguir,” “material” e “Jeffries”. “Seguir” e “material” relacionam-se a acompanhar alguma referência ou utilizar material próprio, enquanto “Jeffries” refere-se à Pamela Jeffries, autora na qual se fundamenta o modelo metodológico proposto neste estudo.

Quantitativamente, seis profissionais mencionaram Pamela Jeffries como referência, três citaram Fabri e seus colaboradores^(16)^, três seguem as recomendações da INACLS^(17)^ e oito utilizaram um instrumento construído por eles ou pela instituição onde trabalham. Três profissionais afirmaram não utilizar nenhum modelo específico. Esses resultados reforçaram a necessidade de elaborar o modelo presente neste artigo.

Na Pergunta 5, a análise textual evidenciou palavras como “especialista”, “validar” e “exemplo”. Alguns profissionais consideram fundamental a participação de especialistas da área de SC, enquanto outros apontam a importância de especialistas no conteúdo do cenário. Quanto à participação do público-alvo no processo de validação, alguns argumentam que a aplicação repetida dos cenários e o público-alvo contribuem para a validação prática, permitindo ajustes conforme são aplicados. A análise por nuvem de palavras revela termos como “especialista” “pensar”, “entender”, “área” e “falar”, enquanto a análise de similitude destaca “profissional”, “aluno” e “validar”.

Da primeira etapa, com revisão teórica e consulta aos especialistas, tem-se a primeira versão do modelo, com 8 categorias e 30 itens. Na segunda etapa, de análise semântica, os dois grupos de avaliadores verificaram se os itens do modelo eram de fácil entendimento. Todos os avaliadores eram enfermeiros e tinham no mínimo o mestrado como última formação (100%; n = 6). Metade dos avaliadores tinham menos de um ano de experiência com a SC (50%; n = 3) e a outra metade possuía experiência acima de um ano. As sugestões dadas nesta etapa resultaram em ajustes textuais de interpretação em 15 dos 30 itens do modelo.

Na validação do conteúdo (n = 6), terceira etapa, participaram quatro especialistas do sexo feminino (67%) com idade entre 30 e 45 anos (67%). Dois participantes tinham mestrado (33%); e quatro, doutorado (67%). Quatro especialistas tinham mais de cinco anos de experiência (67%). A concordância entre juízes, medida pelo IVC, foi calculada nas oito categorias, obtendo-se ao menos 83% de concordância. Cinco categorias alcançaram 100% de concordância em todos os critérios.

Nas categorias “contexto”, “facilitador” e “participante”, houve concordância de 83% no Critério 1 (clareza e objetividade), enquanto as categorias “facilitador”, “estratégias educacionais” e “participante” atingiram 83% nos Critérios 2 (pertinência) e 3 (relevância), conforme Tabela 1. O IVC total resultou em 94% de concordância, indicando validade de conteúdo. Os comentários e sugestões dos peritos foram analisados, resultando em ajustes para maior clareza e aprimoramento do modelo.

**Tabela 1:** Índice de Validade de Conteúdo de cada categoria do modelo completo em função dos critérios de clareza e objetividade, pertinência e relevância

Categoria	IVC		
	Clareza e objetividade %	Pertinência %	Relevância %
Contexto	83	100	100
*Background*	100	100	100
Design	100	100	100
Experiência simulada	100	100	100
Facilitador	83	83	83
Estratégias educacionais	100	83	83
Participante	83	83	83
Resultados	100	100	100
Modelo completo	100	100	100
Média do IVC	94	94	94

*IVC - Índice de Validade de Conteúdo.*

O coeficiente alfa de Cronbach foi calculado para o modelo de forma geral, correlacionando as oito categorias. O resultado foi de 0,886, considerado ótimo. Após as fases de elaboração, análise e validação, obteve-se a versão definitiva destinada à construção de cenários com base na teoria de Jeffries, disponível na seção “Disponibilidade de dados e material” deste artigo e no link https://doi.org/10.48331/scielodata.QTVEUI.

O modelo fornece diretrizes teóricas que embasam conceitualmente a elaboração de cenários e guiam o preenchimento dos 30 itens do modelo. Esses itens devem ser preenchidos nas etapas de planejamento e implementação de cenários. Estão distribuídos em oito categorias, conforme o referencial teórico: Contexto, *Background*, Design, Experiência simulada, Facilitador, Estratégias educacionais, Participante e Resultados. Tais categoriais fundamentam a tomada de decisão e possibilitam a obtenção de resultados satisfatórios.

## DISCUSSÃO

A construção e validação do modelo apresentado neste estudo foram conduzidas com o rigor teórico e metodológico necessário para criar um instrumento fidedigno na condução de planejamentos e implementações de cenários. Esse rigor é essencial para garantir o desenvolvimento de competências, de forma que os cenários reflitam a realidade dinamicamente e se mantenham relevantes em diversos níveis de complexidade^(18)^. A evidência científica subjacente ao planejamento e implementação desses cenários, conforme demonstrado, assegura sua validade, utilidade e replicabilidade, características fundamentais para sua adoção em diferentes ambientes e contextos^(19)^.

A estrutura teórica do modelo, baseada nos conceitos advindos das oito categorias da teoria de Jeffries, demonstrou ser eficaz ao integrar conceitos fundamentais para a prática de SC^(7,20)^. No tocante à categoria “Contexto”, destacou-se a integração curricular ao longo da formação e o contexto em que a SC está inserida, para possibilitar a aplicação de cenários em diferentes níveis de complexidade. Considera-se que contextualizar o cenário - ao ponderar seu meio de inserção, seu contexto específico e o contexto da simulação como um todo - seja crucial no processo de elaboração, de forma que os cenários desenvolvidos sejam relevantes e contribuam para a eficácia da formação^(21,22)^.

Na segunda categoria, “Background”, foi ressaltado no modelo a importância do público alvo, a capacitação prévia do participante e a perspectiva teórica do cenário. A capacitação prévia dos participantes é crucial para alinhar conhecimentos e expectativas, o que, segundo a literatura, resulta em maior retenção e desenvolvimento do aprendizado em SC^(23,24)^. Esses achados reforçam a necessidade de uma preparação robusta antes de executar os cenários, garantindo que os participantes estejam capacitados para enfrentar as situações simuladas.

A categoria “Design” enfatiza a importância da definição planejada dos objetivos para garantir uma experiência simulada efetiva. Os resultados deste estudo e a literatura demonstram que os objetivos não somente orientam a atividade, mas também são fundamentais para garantir que os resultados esperados sejam alcançados^(25,26)^. Além disso, componentes como o *briefing*, o *debriefing*, os papéis dos participantes, o feedback, o realismo e a fidelidade do cenário foram integrados ao modelo, em consonância com a relevância desses termos em estudos recentes^(27,28)^. Infere se, portanto, que eles podem contribuir para replicar a realidade e alcançar resultados efetivos.

O *debriefing,* em particular, foi salientado neste estudo com o objetivo de provocar reflexões críticas nos participantes, de maneira que a prática seja debatida, aprimorada e aconteça tão logo ocorra a execução do cenário^(29,30)^. Os estudos indicam que o facilitador deve ser capacitado para condução do *debriefing*, para que atue como um guia e que os próprios participantes possam fazer conclusões sobre o seu desempenho. O feedback, por sua vez, consiste na correção de eventuais falhas. Neste modelo, recomenda-se que seja feito em formato de dicas, ao longo da condução do cenário, enquanto estudos apontam que o feedback corretivo durante o *debriefing* pode inibir a reflexão e autocrítica^(31)^.

A transição para a categoria “Experiência simulada” traz a interação dinâmica entre facilitador e participante, o ambiente colaborativo centrado no participante e o caráter experiencial do cenário simulado. Esses elementos são apontados como elementos-chave para desenvolver competências, o que trouxe a necessidade de fortalecer os pressupostos básicos da SC no modelo apresentado^(26)^.

Na categoria “Facilitador”, foram exploradas características essenciais do facilitador para o sucesso do cenário. Os resultados e a literatura mostram que a eficácia da simulação depende não apenas da estrutura técnica, mas também das habilidades interpessoais e didáticas do facilitador, que são fundamentais para criar um ambiente de aprendizado seguro e produtivo^(32)^.

Na análise das “Estratégias educacionais”, foi destacado o uso de técnicas centradas no participante, reforçando a importância de uma exposição gradual e repetida a cenários como estratégia eficaz para desenvolver competências^(33)^. Sobre a categoria “Participante”, foram considerados o público-alvo, nível de formação, quantidade de participantes e o papel que eles assumem no cenário. Os dados apoiam a hipótese de que essas abordagens não só aprimoram habilidades, mas também contribuem para a retenção de conhecimento em longo prazo, um fator crucial para a prática clínica e que deve ser considerado antes da implementação de cenários^(34,35)^.

Quanto à categoria “Resultados”, o foco ampliado para incluir os efeitos sobre os pacientes e a relação custo-efetividade para o sistema acrescenta uma nova dimensão à discussão, o que evidencia tanto os benefícios da SC para além do aprendizado dos participantes quanto a evolução do campo da SC^(36,37)^. A correlação entre o desenvolvimento de competências e a melhoria dos resultados clínicos, bem como a redução de custos, sugere que a SC pode ser uma estratégia eficaz tanto do ponto de vista educacional quanto econômico - conclusões, estas, reforçadas pela literatura^(38)^.

Durante a entrevista, termos e conceitos foram acrescentados e implementados no modelo metodológico aqui apresentado, indicando que o processo de construção foi dinâmico e adaptável às necessidades práticas. Além da definição clara dos objetivos, os entrevistados reiteraram que o planejamento é uma etapa fundamental, confirmando a hipótese de que o planejamento é crucial para o sucesso do modelo. Os resultados deste estudo corroboram a literatura, ao destacar o planejamento - etapa mais debatida neste modelo - como indispensável na construção de cenários, garantindo requisitos essenciais para implementação bem-sucedida^(39)^.

A ênfase no levantamento de recursos materiais/humanos e na padronização dos métodos de construção, por checklists e instrumentos de avaliação, mostram esses elementos como cruciais no planejamento, o que foi contemplado neste modelo. Embora alguns recursos sejam caros, a literatura evidencia que não são determinantes para bons resultados^(40)^, demonstrando que a eficácia do modelo depende mais da padronização e da utilização de instrumentos válidos do que dos recursos disponíveis. Quanto à padronização e utilização de instrumentos válidos, observou-se que estes possibilitam avaliação confiável de características da SC, abrangendo habilidades, competências, percepções e desempenho dos participantes, entre outros^(39,41,42)^.

Os entrevistados enfatizaram a relevância da infraestrutura física e dos recursos disponíveis. A literatura aponta que laboratórios de SC bem estruturados e com recursos modernos são vantajosos, especialmente para variação de cenários^(43)^. Embora a qualidade desses laboratórios varie, os dados deste estudo demonstram que a infraestrutura é um elemento importante, mas não obrigatório, para o sucesso da SC. Por exemplo, um estudo realizado na Índia apontou que apenas 44% dos laboratórios avaliados tinham estrutura adequada para simulações^(44)^. Sendo assim, é essencial destacar esses aspectos no design do modelo.

O conhecimento e habilidade dos envolvidos na execução do cenário foi realçado pelos entrevistados, sinalizando que a capacitação dos facilitadores é um fator determinante para a eficácia da SC. A falta de profissionais capacitados é um obstáculo para a disseminação efetiva da SC. Estudo mostra que muitos educadores expressam atitude positiva em relação à SC, mas enfrentam desafios relacionados ao uso de tecnologias e conhecimentos em facilitação^(45)^. Isso indica que há uma premência de investir na formação desses profissionais.

Quando questionados sobre os dificultadores para uso da SC, os entrevistados citaram desafios como a postura profissional, o desenho dos planos pedagógicos dos cursos, a capacitação dos envolvidos e a disponibilidade de recursos e de tempo. Com os resultados, conclui-se que a SC exige abordagem proativa e mudança de mentalidade no tocante às práticas tradicionais. Portanto, o alinhamento aos planos pedagógicos torna-se vital para integrar a SC ao currículo, superar as barreiras de disponibilidade de tempo e garantir abordagem estruturada para alcançar esse objetivo, conforme a literatura evidencia^(22)^.

A capacitação dos envolvidos surge como um fator-chave, corroborando outros estudos que mostram a urgência de investir na formação e qualificação de profissionais para o uso efetivo da SC^(45)^. A falta de recursos financeiros, de infraestrutura tecnológica e de tempo é apontada como uma barreira percebida, ressaltando que é preciso superar desafios logísticos e burocráticos para implementar práticas de SC de alta qualidade^(9)^.

Os resultados deste estudo constatam que a maioria dos entrevistados utiliza um roteiro próprio, o que mostra a importância deste modelo metodológico para fornecer rigor científico e orientar os facilitadores durante a execução dos cenários. A principal hipótese é de que a ausência de roteiros validados baseados em um referencial teórico seja identificada como uma fragilidade no desenvolvimento da SC, tal como reforçam Nascimento e seus colaboradores^(46)^. Ademais, Jeffries foi citada como o referencial mais utilizado, fato que valida a influência significativa da teórica na área.

A validação de cenários por especialistas é reconhecida como uma etapa crítica na elaboração de SC. Essa validação contribui para garantir a confiabilidade, a validação dos objetivos e o aprimoramento do cenário^(47)^. A necessidade de especialistas no tema do cenário em avaliação é destacada pelos entrevistados, a fim de garantir a adaptação à realidade do participante e fundamentação em evidências científicas^(48)^.

A análise semântica visou avaliar se os itens são compreensíveis para diferentes níveis de experiência na população-alvo, ao passo que a validação de conteúdo objetivou assegurar que o conteúdo fosse apropriado e representasse adequadamente os objetivos propostos. A qualificação e experiência encontradas entre os especialistas são destacadas como elementos que conferem qualidade e confiabilidade aos resultados da validação de conteúdo^(19)^.

Ao analisar os resultados, percebe-se que o modelo metodológico desenvolvido atende aos critérios de clareza e objetividade, pertinência e relevância, validados pelos juízes especialistas com concordância superior a 80%, o que reforça sua robustez. A comparação com estudos similares, como checklists e cenários de SC, assim como a discussão aprofundada das categorias fortalecem a validade desse modelo^(42,49,50)^. Os resultados positivos, com IVC global igual a 94% e coeficiente alfa de Cronbach geral de 0,886, apontam uma confiabilidade e concordância significativas entre os especialistas. A ausência de modelos semelhantes na literatura revela a singularidade e inovação do modelo em questão, que não apenas preenche uma lacuna, mas também estabelece novas diretrizes para a prática.

Os resultados deste estudo revelam que o planejamento meticuloso, a capacitação dos envolvidos e a padronização de recursos e processos são fatores críticos para o sucesso da SC. A integração curricular, a superação das barreiras logísticas e a capacitação contínua são essenciais para maximizar o impacto positivo da SC na formação de profissionais de saúde, a fim de garantir não só a eficácia educacional, mas também a melhoria contínua dos cuidados.

### Limitações do estudo

A limitação deste estudo está no reconhecimento relativamente recente da teoria de simulação de Jeffries dentro do panorama da SC, apesar de seu *framework* ser amplamente conhecido. Além disso, sugere-se que o instrumento seja validado com uma amostra superior à apresentada neste estudo, que se limitou às recomendações de análise de conteúdo para construção de instrumentos. Também é importante que futuras validações incluam dados estatísticos mais robustos.

### Contribuições para a área da saúde e enfermagem

O presente estudo visa contribuir com o rigor científico na elaboração e implementação de cenários e facilitar a disseminação da SC, colaborando assim para a formação em saúde, segurança do paciente, melhoria da qualidade da assistência e avanço do conhecimento na área da enfermagem.

## CONSIDERAÇÕES FINAIS

O presente estudo atendeu ao objetivo de desenvolver e validar um modelo metodológico para a construção de cenários, com base na teoria de simulação de Jeffries e na abordagem metodológica rigorosa da psicometria de Pasquali, tornando-se apto para compartilhamento com profissionais da área de SC. Ao disponibilizá-lo à comunidade acadêmica, espera-se contribuir para o rigor científico no processo de elaboração e implementação de cenários, especialmente para enfermagem.

Além disso, espera-se facilitar a disseminação entre docentes/facilitadores com pouca ou nenhuma experiência na área ou entre aqueles com resistência ao uso do método. Com isso, o modelo poderá propiciar a padronização da construção de cenários a fim de permitir ampla utilização em diferentes contextos e eventuais comparações entre estudos.

Acredita-se que a disponibilização desse modelo metodológico para construir cenários de SC possa oferecer uma base orientadora e cientificamente fundamentada para docentes e profissionais facilitadores. O objetivo é garantir que o uso do método alcance seus objetivos, contribuindo para o desenvolvimento de competências de estudantes e de profissionais, para a segurança do paciente e para a melhoria da qualidade da assistência prestada.

## Data Availability

https://doi.org/10.48331/scielodata.QTVEUI
